# D-Dimer Is Associated With Coronary Microvascular Dysfunction in Patients With Non-obstructive Coronary Artery Disease and Preserved Ejection Fraction

**DOI:** 10.3389/fcvm.2022.937952

**Published:** 2022-08-02

**Authors:** Yan Lin, Xiangming Hu, Weimian Wang, Bingyan Yu, Langping Zhou, Yingling Zhou, Guang Li, Haojian Dong

**Affiliations:** ^1^Shantou University Medical College, Shantou, China; ^2^Department of Cardiology, Guangdong Cardiovascular Institute, Guangdong Provincial Key Laboratory of Coronary Heart Disease Prevention, Guangdong Provincial People’s Hospital, Guangdong Academy of Medical Sciences, Guangzhou, China; ^3^The Second School of Clinical Medicine, Southern Medical University, Guangzhou, China; ^4^School of Medicine, South China University of Technology, Guangzhou, China

**Keywords:** non-obstructive coronary artery disease, preserved ejection fraction, coronary microvascular dysfunction, TIMI myocardial perfusion grade, D-dimer

## Abstract

**Background:**

Coronary microvascular dysfunction (CMVD), an important etiology of ischemic heart disease, has been widely studied. D-dimer is a simple indicator of microthrombosis and inflammation. However, whether an increase in D-dimer is related to CMVD is still unclear.

**Materials and Methods:**

This retrospective study consecutively enrolled patients with myocardial ischemia and excluded those with obstructive coronary artery. D-dimer was measured at admission and the TIMI myocardial perfusion grade (TMPG) was used to distinguish CMVD. Patients were divided into the two groups according to whether the D-dimer was elevated (>500 ng/ml). Logistic models and restricted cubic splines were used to explore the relationship between elevated D-dimer and CMVD.

**Results:**

A total of 377 patients were eventually enrolled in this study. Of these, 94 (24.9%) patients with CMVD had older age and higher D-dimer levels than those without CMVD. After full adjustment for other potential clinical risk factors, patients with high D-dimer levels (>500 ng/ml) had a 1.89-times (95% CI: 1.09–3.27) higher risk of CMVD than patients with low D-dimer levels. A non-linear relationship was found between concentrations of D-dimer and CMVD. With increased D-dimer level, the incidence of CMVD increased and then remained at a high level. Stratified analysis was performed and showed similar results.

**Conclusion:**

Elevated D-dimer level is associated with the incidence of CMVD and potentially serves as a simple biomarker to facilitate the diagnosis of CMVD for patients with angina.

## Introduction

Although clinical practice strategies have optimized the prevention and treatment of ischemic heart disease over the past few years, ischemic heart disease has a complex pathophysiology that goes beyond the traditional role of obstructive coronary artery disease (CAD). Coronary microvascular dysfunction (CMVD), a phenotype of ischemic heart disease, is defined as the clinical syndrome of angina without obstructive CAD (1). CMVD may contribute to angina by reducing coronary blood flow, which is prevalent and associated with an increased risk of future adverse cardiovascular outcomes (2, 3). Studies have shown that CMVD is mediated by risk factors traditionally recognized as related to cardiovascular disease, although these factors account for a small sample, leaving a large proportion that cannot be explained (4, 5). Potential pathophysiological mechanisms of ischemia involved in CMVD with non-obstructive CAD have been previously proposed such as microvascular thrombosis, inflammation, and edema (6), indicating a changed myocardial microcirculation environment. However, there is currently a lack of simple tools to identify CMVD and more predictors are still desperately needed.

D-dimer, a degradation product of cross-linked fibrin, is widely recognized as a marker of thrombosis (7). Elevated D-dimer has a prognostic value for adverse cardiovascular events in healthy people and patients with CAD (8, 9). Zhang et al. found that D-dimer in admission could be used to identify the no-reflow phenomenon in patients with ST-segment elevation myocardial infarction, highlighting the advantage of D-dimer in identifying microvascular embolism (10). Previous studies have shown that elevated D-dimer levels are related to microvascular thrombosis (11), inflammation (12), and endothelial injury (13), which contribute to CMVD. The TIMI myocardial perfusion grade (TMPG) is a simple indicator derived from coronary angiography to evaluate coronary microcirculation and identify CMVD (14–16). Although D-dimer is a simple indicator of the microcirculatory environment, the relationship between D-dimer level and CMVD has only rarely been studied.

In this study, we hypothesized that D-dimer levels might be associated with CMVD evaluated by the TMPG and could be used as an available biomarker for early screening for CMVD.

## Materials and Methods

### Study Population

**FIGURE 1:**
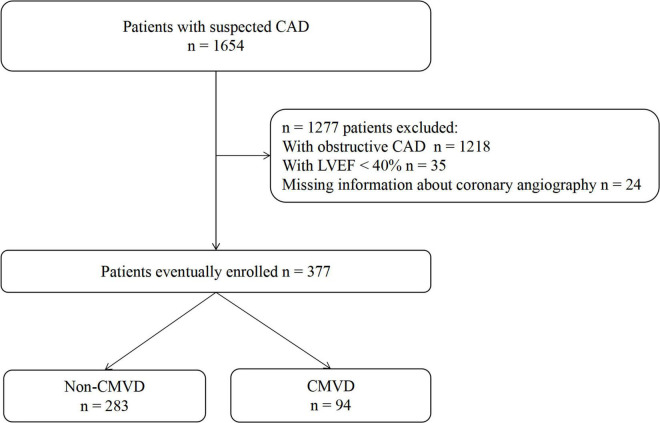
Study flowchart.

This retrospective study included 1,654 consecutive patients who were admitted for suspected CAD from September 2014 to September 2015 at the Guangdong Provincial People’s Hospital. Suspected CAD is clinically based on symptoms of ischemia and/or electrocardiographic ischemic changes. Patients with obstructive coronary artery stenosis (defined as ≥70% luminal diameter narrowing of an epicardial stenosis or ≥50% luminal diameter narrowing of the left main artery, *n* = 1,218), with impaired left ventricular ejection fraction (LVEF) (<40%, *n* = 35) and without coronary angiography data (*n* = 24) were excluded (Figure 1). Demographic data, risk factors, and coronary angiography results were collected based on the electronic medical records.

This study was approved by the Ethics Committee of Guangdong Provincial People’s Hospital and informed verbal consent was obtained from all the patients. This research was conducted in accordance with the Declaration of Helsinki.

### Coronary Angiography

All the patients underwent coronary angiography using the Judkins technique. Coronary angiography was performed with a radial approach and the femoral artery was used in a minority of patients, as clinically necessary. We used 5-Fr or 6-Fr Judkins left and right diagnostic catheters for left and right coronary angiography, respectively. The degree of coronary artery stenosis was judged and recorded by two interventional cardiologists. Evaluation of the TMPG flow was performed by two experienced cardiologists who were blinded to patient’s demographic and clinical information. In the case of disagreement, a third cardiologist was consulted and the majority opinion was adopted.

The TMPG was classified into four grades (17) as follows: (1) TMPG 0: failure of dye to enter the microvasculature, indicating a lack of tissue-level perfusion; (2) TMPG 1: dye enters slowly but fails to exit the microvasculature. There is a ground-glass appearance or opacification of the myocardium in the distribution of the vessel that fails to clear from the microvasculature and dye staining is present on the next injection (30 s); (3) TMPG 2: delayed entry and exit of dye from the microvasculature. Dye strongly persists after three cardiac cycles of the washout phase and either does not or only minimally diminishes in intensity during washout; and (4) TMPG 3: normal entry and exit of dye from the microvasculature. Dye is gone or is mildly/moderately persistent after three cardiac cycles of the washout phase and noticeably diminishes in intensity during the washout phase. The blush that is of only mild intensity throughout the washout phase but fades minimally is also classified as grade 3.

### Definitions and Laboratory Examination

The TMPG flow was used to assess coronary microvascular function and CMVD was distinguished with the TMPG flow <3. Hypertension, diabetes, chronic kidney disease (CKD), smoking, and alcohol consumption were diagnosed according to self-report and discharge diagnosis. A blood routine was detected using the Sysmex XE-5000 machine. High-density lipoprotein cholesterol (HDL-C), low-density lipoprotein cholesterol (LDL-C), total cholesterol, triglyceride, lipoprotein(a), albumin, uric acid, and creatinine were detected using the Beckman AU5800 spectrophotometer *via* colorimetry or immunoturbidimetry. D-dimer was detected using the Sysmex CA-1500 *via* immunoturbidimetry.

### Echocardiographic Analysis

Transthoracic echocardiography examination was performed by experienced sonographers using the GE Vivid E95 (GE Healthcare, Milwaukee, WI, United States) interfaced with a 2.5–3.5-MHz phased array probe. LVEF was measured using Simpson’s method.

### Statistical Analysis

The total procedure for statistical analysis was divided into four steps. First, we used the *t*-test for normally distributed data, the Mann–Whitney *U* test for non-normally distributed data, and the Chi-squared test or Fisher’s exact test for categorical variables to identify significant differences between the two groups. Second, we used the logistic regression models simultaneously for unadjusted, minimally adjusted, and fully adjusted analyses to evaluate the associations between D-dimer and CMVD. Considering the potential influence of age on D-dimer concentration, we added the sensitivity analyses to assess the relationship using an age-adjusted cut-off (500 ng/ml, if age is <50 years or age in years ×10 in patients ≥50 years) (18, 19). Third, because D-dimer is a continuous variable, to visually assess the non-linear relationship between D-dimer level and risk of CMVD, a restricted cubic spline curve was used. Fourth, subgroup analyses were performed using stratified Chi-square models; interactions among subgroups were examined using likelihood ratio tests. Comparisons with *P* < 0.05 (two-sided) were considered to be statistically significant. All of the analyses were performed with Stata 15.0 (StataCorp LLC, College Station, TX, United States), R version 3.4.3 (The R Project for Statistical Computing, Vienna, Austria), and EmpowerStats (X&Y Solutions Incorporation, Boston, MA, United States).

## Results

### Patient Characteristics

A total of 377 patients who presented with symptoms of ischemia changes and without obstructive CAD were finally included. Of those, 94 patients had evidence of CMVD assessed using the TMPG flow. The clinical characteristics of patients with CMVD and non-CMVD are shown in Table 1. Patients with CMVD were more likely to be older, have more frequent smoking behavior, and have more hypertension than those without patients without CMVD. D-dimer levels were significantly higher in patients with CMVD than in controls [360.00 (270.00–747.50) vs. 330.00 (270.00–470.00)], while sex, alcohol consumption, diabetes, renal function, blood lipids, and previous medication use presented no difference between the two groups.

**TABLE 1:** Demographic and clinical characteristics of patients included in the study.

	Non-CMVD	CMVD	*p*-value
	*n* = 283	*n* = 94	
Age, years	61 ± 11	64 ± 9	0.043
Male sex	150 (53.00%)	56 (59.57%)	0.268
Current smoking	64 (22.61%)	31 (32.98%)	0.045
Alcohol consumption	16 (5.65%)	7 (7.45%)	0.529
Hypertension	134 (47.35%)	65 (69.15%)	< 0.001
Diabetes	50 (17.67%)	19 (20.21%)	0.580
CKD	30 (10.60%)	11 (11.70%)	0.766
WBC, 10^9^/L	7.20 ± 2.04	7.33 ± 1.88	0.269
Platelets, 10^9^/L	223.42 ± 61.03	216.52 ± 66.36	0.354
Hemoglobin, g/L	132.50 ± 15.33	134.86 ± 16.28	0.203
Glucose, mmol/L	5.61 ± 1.58	5.86 ± 2.37	0.248
TG, mmol/L	1.58 ± 1.25	1.66 ± 1.09	0.601
TC, mmol/L	4.62 ± 1.24	4.55 ± 1.01	0.615
LDL-C, mmol/L	2.68 ± 1.08	2.61 ± 0.87	0.587
HDL-C, mmol/L	1.16 ± 0.29	1.15 ± 0.27	0.783
Creatine, μmol/L	78.20 ± 27.60	77.72 ± 22.31	0.877
D-dimer, ng/mL	330.00 (270.00–470.00)	360.00 (270.00–747.50)	0.028
CRP, mg/L	1.52 (0.55–4.35)	1.64 (0.57–3.58)	0.669
LVEF, %	66 (62–69)	65 (61–70)	0.383
**Previous drug treatment**			
ACE inhibitor or ARB, %	44 (15.55%)	14 (14.89%)	0.879
Beta-blocker, %	42 (14.84%)	10 (10.64%)	0.306
Calcium channel blocker, %	30 (10.60%)	15 (15.96%)	0.165
Diuretics, %	7 (2.47%)	1 (1.06%)	0.685
Statin, %	50 (17.67%)	19 (20.21%)	0.580

*CMVD, coronary microvascular dysfunction; CKD, chronic kidney disease; WBC, white blood cells; TG, triglyceride; TC, total cholesterol; LDL-C, low-density lipoprotein-cholesterol; HDL-C, high-density lipoprotein-cholesterol; CRP, C-reactive protein; LVEF, left ventricular ejection fraction; ACE, angiotensin converting enzyme; ARB, angiotensin receptor blocker.*

### Association Between D-Dimer and Coronary Microvascular Dysfunction

Results for the association between D-dimer and CMVD are shown in Table 2. In the unadjusted model, the odds ratio (OR) for CMVD of D-dimer was 2.12 (95% CI: 1.29–3.50). After fully adjusting for other potential clinical risk factors, including age, sex, hypertension, diabetes, smoking, alcohol consumption, and platelets, the risk of CMVD among those with high D-dimer levels (>500 ng/ml) was 1.86-times (95% CI: 1.09–3.19) higher than the risk among patients in low D-dimer levels (*P* < 0.05). The association between the age-adjusted D-dimer and CMVD remained remarkably significant.

**TABLE 2:** Association of CMVD and D-dimer in study participants.

Variable	Model 1*^†^*	Model 2*^†^*	Model 3*^†^*	Model 4*^†^*
**D-dimer was converted into a binary variable according to a cutoff of >500 ng/mL**				
OR	2.12	1.91	1.88	1.86
95% CI	1.29–3.50	1.13–3.22	1.10–3.23	1.09–3.19
Chi-square (DF)	8.49 (1)	11.91 (3)	25.11 (7)	26.31 (8)
P-value	0.003	0.016	0.021	0.024
**D-dimer was converted into a binary variable according to age-related cutoff value**				
OR	2.25	2.06	1.95	1.92
95% CI	1.29–3.91	1.17–3.62	1.10–3.48	1.08–3.43
Chi-square (DF)	7.95 (1)	12.40 (3)	24.87 (7)	26.00 (8)
P-value	0.004	0.012	0.023	0.027

*^†^Model 1: Unadjusted. Model 2: Adjusted for age and sex. Model 3: Adjusted as in Model 2 and hypertension, diabetes, smoking, and alcohol consumption. Model 4: Adjusted as in Model 3 and platelets.*

*CMVD, coronary microvascular dysfunction. OR, odds ratio; CI, confidence interval; DF, degrees of freedom.*

**FIGURE 2:**
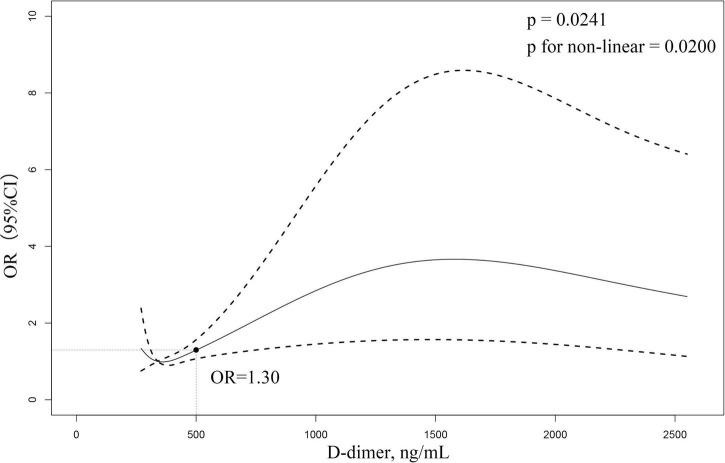
Restricted cubic spline curve to fit the relationship between D-dimer level and CMVD. The model adjusted for age, sex, hypertension, diabetes, smoking, alcohol consumption, and platelets. The middle area of the dash represents 95% confidence interval (CI), and the reference line represents D-dimer margin level. The relationship between D-dimer level and CMVD is not shown for those D-dimer >2,500 ng/mL due to the large (95%) CI.

The relationship between D-dimer and the risk of CMVD is given in Figure 2. The restricted cubic spline curve showed a non-linear relationship between the D-dimer level and the prevalence of CMVD (*P* for linearity <0.05). An elevated D-dimer level was significantly associated with an increased risk of CMVD (*P* = 0.0241). When the D-dimer was at a margin level (500 ng/ml), the OR for CMVD was 1.30.

### Subgroup Analyses

**FIGURE 3:**
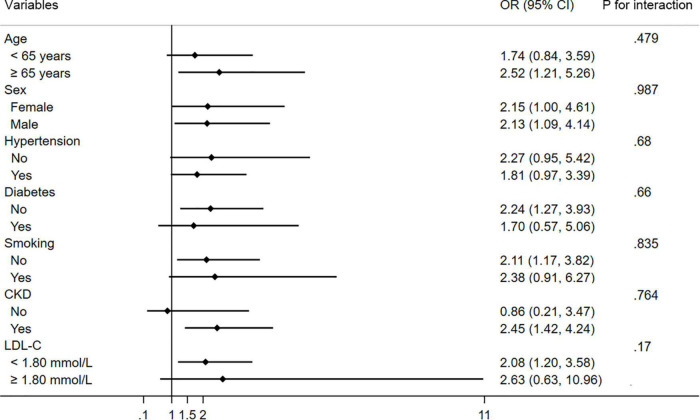
Subgroup analysis of the association between CMVD and D-dimer in prespecified and exploratory subgroups. CI, confidence interval.

In subgroup analysis, patients with high D-dimer levels (>500 ng/ml) have CMVD more frequently than those without in most of the strata (Figure 3). No significant interactions for the association between D-dimer and CMVD were found among individuals stratified by age, sex, hypertension, diabetes, smoking, CKD, and LDL-C levels.

## Discussion

In the present study, we found that elevated D-dimer level was strongly associated with the risk of CMVD and the association was independent of traditional risk factors. There was a dose–response relationship between D-dimer concentration and the incidence of CMVD. Elevated D-dimer levels could be used as a biomarker to identify CMVD.

D-dimer, a biomarker of microthrombosis, has been previously reported to reflect microcirculation (11). Previous studies have shown that elevated D-dimer levels are associated with cerebral circulation and microvascular complications in patients with diabetes mellitus, indicating the role of D-dimer in evaluating microvascular function (20, 21). However, there has been little study on the correlation between D-dimer and coronary microcirculation. In our study, the concentration of D-dimer was higher in patients with CMVD than in those without CMVD. Elevated D-dimer levels had a significant relationship with the occurrence of CMVD and the non-linear relationship between them also supports it. In the general population and patients with CAD, elevated baseline D-dimer levels were associated with poor cardiovascular outcomes (8, 9, 22), which may be attributed to the presence of CMVD. In addition, there is also an individual susceptibility to the occurrence of CMVD (23). Acquired risk factors such as older age, hypertension, diabetes, and hyperlipidemia may increase the occurrence of CMVD. In our study, patients with high D-dimer levels were older, had a higher incidence of hypertension, and were more likely to smoke. These comorbidities might contribute to CMVD.

The mechanisms by which D-dimer reflects microcirculation mainly include microvascular thrombosis, endothelial dysfunction, and inflammation. Previous studies on CMVD after ST-segment elevation myocardial infarction have shown that elevated D-dimer levels were largely influenced by thrombus burden and distal microvascular thrombosis (10). In addition to distal microvascular embolization, Erkol et al. found that *in situ* thrombosis may also contribute to poor myocardial perfusion (24). D-dimer, a biomarker that can reflect the severity of hypercoagulability, can be increased by microvascular embolism. Research involving patients with angina, non-obstructive CAD, and normal left ventricular function has shown that endomyocardial biopsy-proven endothelial cell activation occurred more frequently in patients with CMVD (25). Normal coronary blood flow and myocardial perfusion rely on the normally functioning endothelium, which regulates smooth muscle function through the release of vasodilators, such as nitric oxide (26). Inversely, damage to endothelial cells would lead to platelet activation and a coagulation cascade (27). The correlation between elevated D-dimer and endothelial cell dysfunction has been widely reported in previous studies (28–30). The significantly independent association between D-dimer levels and endothelial function determined *via* flow-mediated dilatation of the brachial artery supports the role of D-dimer as a biomarker for endothelial dysfunction (31), suggesting that an increased D-dimer level indicates the presence of endothelial dysfunction. Another potential mechanism contributing to CMVD is vascular inflammation. Prior studies have suggested that traditional cardiovascular risk factors may be important and they fail to fully account for the increased risk of the development of CMVD (32). In this regard, Klein et al. reported that patients with biopsy-proven myocardial inflammation infiltrate had an apparently reduced coronary flow reserve, indicating an association between inflammation and the occurrence of microvascular dysfunction (33). What is more, high-sensitivity C-reactive protein (CRP) is inversely related to coronary flow reserve in patients with angina and without obstructive CAD, showing a direct relationship between inflammation and CMVD (34). D-dimer, as an acute phase reactant, is increasingly recognized as a marker of inflammatory reaction (27). D-dimer concentrations have been reported to be related to vascular inflammation and microvascular complications in patients with metabolic and infectious diseases, such as diabetes mellitus, chronic obstructive pulmonary disease, and HIV infection (10, 35). Our results indicated that inflammation markers, such as white blood cell count and CRP, were slightly higher in patients with CMVD, although not significantly. In all, the microenvironment of inflammation and endothelial damage reflected by D-dimer plays an important role in the development of CMVD among patients with ischemic heart disease.

This study has several limitations. First, the present study had a retrospective cross-sectional design, so no causal relationship can be inferred between D-dimer levels and CMVD. Second, the research was conducted in a single center and the sample size is relatively small, so the conclusions drawn here cannot be generally extrapolated. Third, other factors that influence the level of D-dimer were not considered in this study, including infectious diseases, pulmonary embolism, and subclinical deep vein thrombosis. However, the effect of these factors can be ruled out because individuals with highly abnormal D-dimer levels were excluded and the vast majority of the study population had D-dimer concentrations of <2,500 ng/ml. Fourth, it is widely recognized that the TMPG is a clinically accessible and practical method to assess CMVD. However, other more accurate quantitative methods could be considered as options for further studies, such as cardiac MR, index of microcirculatory resistance, and fractional flow reserve.

## Conclusion

In summary, D-dimer levels are significantly associated with the incidence of CMVD. This biomarker may be useful in identifying patients with CMVD for ischemic heart disease.

## Data Availability Statement

The raw data supporting the conclusions of this article will be made available by the authors, without undue reservation.

## Ethics Statement

The studies involving human participants were reviewed and approved by the Ethics Committee of Guangdong Provincial People’s Hospital. Written informed consent for participation was not required for this study in accordance with the national legislation and the institutional requirements.

## Author Contributions

XH, YL, and HD conceived and designed study, and drafted and refined the manuscript. WW, BY, LZ, and XH collected and complied, and analyzed the data. YZ, GL, and HD revised the manuscript critically. All authors have read and agreed to the published version of the manuscript.

## Conflict of Interest

The authors declare that the research was conducted in the absence of any commercial or financial relationships that could be construed as a potential conflict of interest.

## Publisher’s Note

All claims expressed in this article are solely those of the authors and do not necessarily represent those of their affiliated organizations, or those of the publisher, the editors and the reviewers. Any product that may be evaluated in this article, or claim that may be made by its manufacturer, is not guaranteed or endorsed by the publisher.
